# Ratiometric Sensor Based on PtOEP-C6/Poly (St-TFEMA) Film for Automatic Dissolved Oxygen Content Detection

**DOI:** 10.3390/s20216175

**Published:** 2020-10-29

**Authors:** Honglin Zhang, Zhiguo Zhang

**Affiliations:** 1 School of physics, Harbin Institute of Technology, Harbin 150001, China; zpp13125@163.com; 2 School of Instrumentation Science and Engineering, Harbin Institute of Technology, Harbin 150001, China

**Keywords:** ratiometric oxygen sensor, optical parameter, detection limit, DO kinetic process

## Abstract

A ratiometric oxygen sensor based on a platinum octaethylporphyrin (PtOEP)–coumarin 6 (C6)/poly (styrene-trifluoroethyl methacrylate) (poly (St-TFEMA)) film was developed for automatic dissolved oxygen (DO) detection. The oxygen-sensing film according to the dynamic quenching mechanism was prepared by embedding platinum octaethylporphyrin (PtOEP) and coumarin 6 (C6) in poly (styrene-trifluoroethyl methacrylate) (poly (St-TFEMA)). The optical parameter (*OP*) was defined as the ratio of the oxygen-insensitive fluorescence from C6 to the oxygen-sensitive phosphorescence from PtOEP. A calibration equation expressing the correlation between the *OP* values and DO content described by a linear function was obtained. A program based on the Labview software was developed for monitoring the real-time DO content automatically. The influence of the excitation intensity and fluctuation on the *OP* values and the direct luminescence signal (integration areas) was compared, verifying the strong anti-interference ability of the sensor. The detection limit of the sensor was determined to be 0.10 (1) mg/L. The switching response time and recovery time of the sensor were 0.4 and 1.3 s, respectively. Finally, the oxygen sensor was applied to the investigation of the kinetic process of the DO content variation, which revealed an exponential relationship with time.

## 1. Introduction

Oxygen is an essential element for the survival of human beings, animals, and plants, as well as for social development [1,2]. Oxygen content measurement is of great significance in environmental monitoring, clinical medicine, aquaculture, the food industry, and marine science [3]. In the food industry, the presence of dissolved oxygen (DO) directly affects the taste of beer and other products [4]. Serious water pollution due to organic pollutants [5] results in a lower DO value. The traditional Winkler titrimetric analysis method [6] and Clark electrode method [7] exhibit high accuracy in the measurement of DO content; however, the detection process is oxygen-consuming and seriously interfered with by external magnetic fields and organic solvents or gas [8,9]. To compensate for the deficiencies of the above two methods, researchers have developed optical oxygen sensors according to the principle of dynamic fluorescence quenching [10,11,12] for DO monitoring, which present excellent features including high sensitivity and accuracy, long-term stability, good anti-electromagnetic interference, and a lack of oxygen consumption [13,14].

Currently, the widely used method for DO measurement is dependent on a single wavelength luminescence intensity, and is simple and easy to operate, accompanied by mature technology and testing instruments [15]. However, the detection signal can be seriously affected by the stability of the light source, probe concentration distribution [16], and optical path system [17]. In recent years, the ratiometric oxygen sensor [18,19] based on a dual wavelength luminescence emission mechanism has been developed for accurate DO detection, receiving an increasing amount of attention. Ratiometric oxygen-sensing technology could overcome the issues caused by the single wavelength method, correct the influence of the probe concentration distribution, and eliminate the system error effectively [20,21]. Generally, the ratiometric oxygen sensor is immobilized an oxygen-sensitive indicator and an oxygen-insensitive reference indicator on appropriate matrix materials [19]. The structural design and performance optimization of the sensor is mainly dependent on the choice of indicators, the matrix, and their interactions. Chen et al. [22] prepared a ratiometric oxygen-sensing film by using PtF_20_TPP as the oxygen-sensitive probe and CdTe quantum dots as the reference probe, which can be applied for fast DO detection with a 0.5 mg/L detection limit. Zhao et al. [23] developed a ratiometric oxygen-sensing system based on phosphorescence from Gd-HMME and fluorescence from filter paper, which was limited to only gaseous oxygen detection owing to the irreducible pollution of the water environment that was evident with dissolved oxygen detection. Although much research has been conducted in recent years, the current oxygen sensor is still limited to single-factor detection. Thus, it is particularly important to develop an oxygen sensor with good oxygen-detection capability and strong anti-photo stability. Meanwhile, the sensor can be recycled, avoiding irreversible pollution of the measured environment.

In this study, we aimed to develop a PtOEP-C6/Poly(St-TFEMA) ratiometric oxygen sensor with high oxygen sensitivity, strong anti-interference ability, and accurate detection capabilities. Herein, PtOEP and C6 were selected as oxygen-dependent and oxygen-independent indicators, respectively. The optical parameter (*OP*) was the ratio of the luminescence integral area of C6 to that of PtOEP, and the calibration equation between *OP* and DO is obtained The ratiometric oxygen sensor was developed based on the Labview software to achieve the automatic measurement of the DO content. The performance parameters including the detection limit, relative uncertainty, response time, fluctuations, and *OP* stability were evaluated. Finally, the oxygen sensor was utilized for the study of the DO kinetics in water samples. The developed oxygen sensor can be expected to be applied in the fields of food fermentation and sewage treatment or biomedical field in the future by monitoring the change in oxygen content in real time, so as to achieve the accurate detection and strict control of oxygen content.

## 2. Experimental Section

### 2.1. Materials

Platinum octaethylporphine (PtOEP) and coumarin 6 (C6) were obtained from J&K Chemical Co., Ltd., (Shanghai, China) and 2,2-Azobisisobutyronitrile (AIBN), Styrene (St), and trifluoroethyl methacrylate (TFEMA) were supplied by Alfa Aesar Co., Ltd., (Shanghai, China). Toluene was provided by Xilong Reagent Co., Ltd., (Shantou, China) Anhydrous magnesium sulphate (MgSO_4_) was obtained from Tianjin Kermel Chemical Co., Ltd., (Tianjin, China). High-purity nitrogen and oxygen were purchased from Harbin Liming Co., Ltd., (Harbin, China). The AIBN was dried after re-crystallization from ethanol. The St and TFEMA were washed with 5% NaOH to remove the inhibitor and dried with anhydrous MgSO_4_.

### 2.2. Preparation of the Ratiometric Oxygen-Sensing Film

The fluoropolymer of poly (St-TFEMA) was synthesized via the solvothermal method. First, the polymer precursor was obtained by dissolving a mixture of St (50 mmol), TFEM (50 mmol), and AIBN (1.0% total mass of monomer) in toluene (15 mL). Then, the mixture solution was transferred into the reactor, and the polymerization reaction was allowed to proceed at 80 °C and continuously stirred at 300 rpm for 7 h under the protection of a nitrogen atmosphere. Next, the polymer solution was mixed with the PtOEP/toluene (1 mM) and C6/toluene (0.5 mM) indicator solution in a volumetric ratio of 10:1. Finally, the PtOEP-C6/Poly(St-TFEMA) ratiometric sensing film was obtained by casting the mixture solution onto quartz glass by using a four-side coating device (thickness controllable) and then stored in a dark place.

### 2.3. Instruments and Characterization

The ultraviolet–visible spectrum was used to study the absorption properties of PtOEP and C6. During the test process of UV–Vis absorption, a deuterium lamp was selected as the light source and an optical spectrometer (Ocean Optics QE65000) was the detecting device. The oxygen-sensing performance of the PtOEP-C6/Poly (St-TFEMA) ratiometric oxygen-sensing film was studied with a fiber-optic spectrometer (Ocean Optics USB2000, integration time = 100 ms, average time = 20) equipped with a diode laser centered at 405 nm as the excitation source. Furthermore, a nitrogen oxygen mixture device equipped with a gas flow-meter was used to obtain environments with different dissolved oxygen concentrations, and a commercial dissolved oxygen meter was used to calibrate the actual content.

### 2.4. Automatic Ratiometric Oxygen-Sensing System

**Figure 1:**
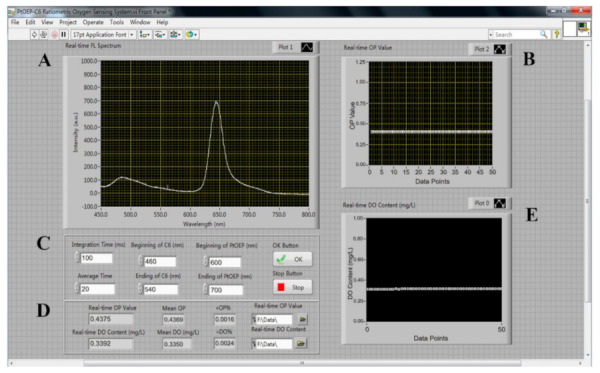
The front panel of the automatic ratiometric sensing system.

A ratiometric oxygen sensor based on the Labview software was developed to achieve the automatic measurement of the DO content. Figure 1 shows the front panel of the ratiometric oxygen-sensing system.

The program Panel Area A displays the online luminescence emission spectrum recorded by the USB2000 spectrometer.

Panel Area B exhibits the real-time *OP* values.

The program Panel Area C is used to set the parameters including the integration time, average time, and beginning and ending wavelength of PtOEP and C6. Meanwhile, the OK and Stop buttons were used to start and shut down the system.

The program Panel Area D is for the file management and data analysis processes, displaying the real-time value and deviation value of the *OP* and DO content.

Panel Area E indicates the DO content calculated from the measured *OP* values combined with the *OP*–DO calibration equation.

## 3. Results and Discussion

### 3.1. Optical Properties of PtOEP and C6 Indicator

**Figure 2:**
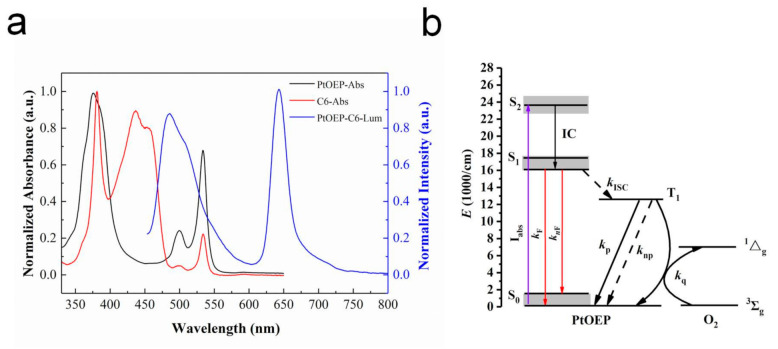
(**a**) Typical normalized UV-absorption spectrum (platinum octaethylporphyrin (PtOEP)—black; coumarin 6 (C6)—red) and luminescence spectrum (blue) of the PtOEP/toluene and C6/toluene solutions; (**b**) The energy level structure of PtOEP.

In this study, PtOEP was chosen as the oxygen-quenchable indicator and C6 as the reference indicator that was insensitive to oxygen. The typical normalized UV-absorption spectrum (PtOEP—black; C6—red) and luminescence spectrum (blue) of the PtOEP/toluene and C6/toluene solutions are presented in Figure 2a and were used to determine the maximum emission wavelength of the indicators. The UV spectrum indicates that PtOEP has a strong Soret band centered at 378 nm and two weaker Q-bands located at 499 and 534 nm; C6 comprises a Soret band centered at 382 nm accompanied by four Q-bands located at 437, 454, 501 and 533 nm. The excited state of PtOEP exhibits two phosphorescence emission peaks at 645 and 711 nm. C6 has two strong fluorescence emission peaks at 484 and 510 nm. The strong luminescence peaks at 645 nm of PtOEP and 484 nm of C6 were selected for the following study.

Accordingly, the energy level structure of PtOEP is shown in Figure 2b. The particles of PtOEP in the ground state S_0_ are pumped into the singlet excited state S_2_, and then transferred to the excited state S_1_ with lower energy through internal conversion (IC). The particles in S_1_ return to S_0_ via fluorescence emission (*k*_F_) and non-radiative relaxation (*k*_nF_); meanwhile, a certain number of particles reach the triplet excited state T_1_ through Intersystem Crossing (ISC) [24,25]. However, T_1_ to S_0_ are transition forbidden; there are two energy transfer pathways for T_1_-state particles: returning to S_0_ by non-radiative relaxation and emitting phosphorescence. Phosphorescence quenching occurs when oxygen molecules exist in the surrounding environment, and the phosphorescence emission energy is plundered by oxygen molecules, which leads to the decay of the phosphorescence intensity or lifetime [23].

### 3.2. Ratiometric Oxygen-Sensing Properties and the Calibration Curve

**Figure 3:**
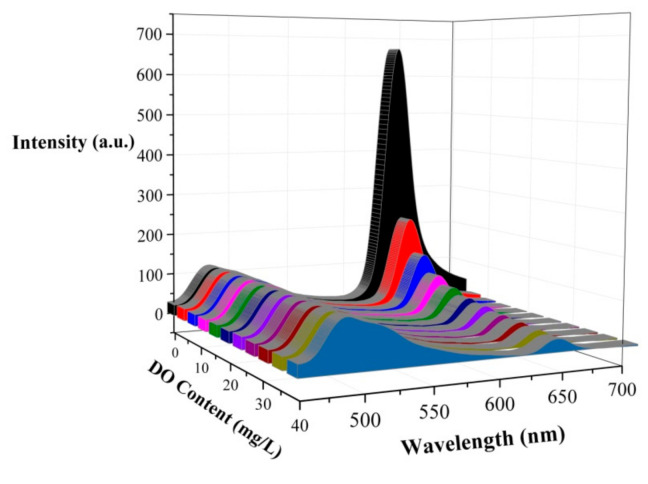
Variation in the luminescence intensity of the ratiometric sensing film with dissolved oxygen (DO) content.

The variation of the luminescence intensity of the PtOEP-C6/Poly (St-TFEMA) ratiometric sensing film with the DO content is shown in Figure 3. The phosphorescence intensity of PtOEP decreases dramatically with DO content, which indicates that the phosphorescence of the PtOEP-C6/Poly (St-TFEMA) film at 645 nm can be quenched by oxygen molecules effectively in the surrounding environment. Meanwhile, the reference indicator C6 exhibits a stable fluorescence signal at 484 nm. Hence, the satisfactory ratiometric oxygen-sensing properties of the sample were verified.

**Figure 4:**
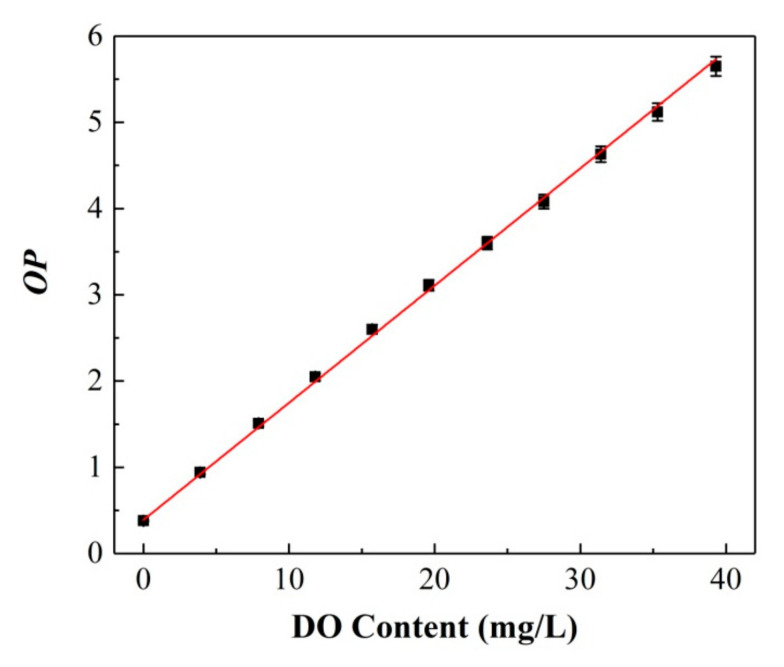
Calibration curve between the optimal parameter (*OP*) value and DO content of the ratiometric sensing film.

Area 1 of C6, ranging from 460 to 540 nm, was selected as the reference signal, and the integral area of S_1_ was only proportional to the pump light intensity *I*_pump_. Area 2 of PtOEP, ranging from 600 to 700 nm, was the research signal, and the integral area of S_2_ was not only related to the pump intensity *I*_pump_ but also greatly dependent on the variation in the oxygen concentration. Herein, the optical parameter *OP* was defined as S_1_/S_2_, which only related to the oxygen concentration *f*([O_2_]). Figure 4 presents the measured *OP* value versus the increase in oxygen concentration, and the corresponding calibration curve is expressed as follows:

(1) $$OP=S_{1}/S_{2}=f\left(\left[DO\right]\right)\;=0.39+0.14\left[DO\right]$$

It can be seen that the value of OP exhibits a correlation with the DO content described by a linear function Calibration standard deviation (RMSEC = 1.73%). Moreover, all the data points were obtained by collecting 20 points in the same measurement process (the same later) to verify the repeatability of the calibration curve. Therefore, the DO content for any unknown sample can be calculated combined with the above calibration equation by measuring the *OP* values.

### 3.3. Anti-Interference Ability of the Ratiometric Oxygen Sensor

**Figure 5:**
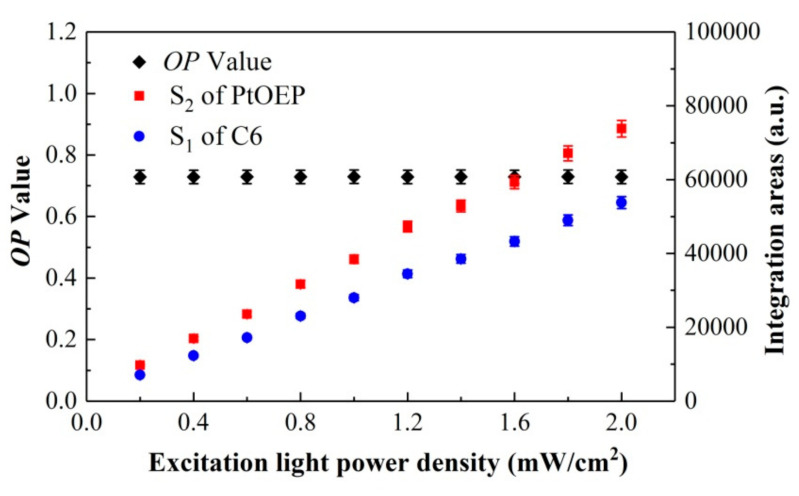
Variations in *OP*, S_2_ of PtOEP, and S_1_ of C6 versus excitation light power density.

Theoretically, the ratiometric method can effectively eliminate some interference factors during the testing process, such as the variation in the excitation light power density or the indicator concentration distribution [26,27,28]. To verify that the actual measurement value of the *OP* in this system was unrelated with the variation and fluctuation of the excitation intensity, the influence of the excitation power density *I*_pump_ on the *OP* values was investigated in detail. Figure 5 shows the variation in the *OP* values, S_2_ of PtOEP, and S_1_ of C6 versus the excitation light power density *I*_pump_. The test results indicate that the directly measured integration areas S_2_ of PtOEP and S_1_ of C6 increase monotonously with the increase in the excitation light power density *I*_pump_, whereas the value of *OP* based on the ratiometric method remains stable with the increase in *I*_pump_. Therefore, it can be illustrated that the ratiometric oxygen sensor can effectively eliminate the interference of the variation of the excitation light during the measurement.

**Figure 6:**
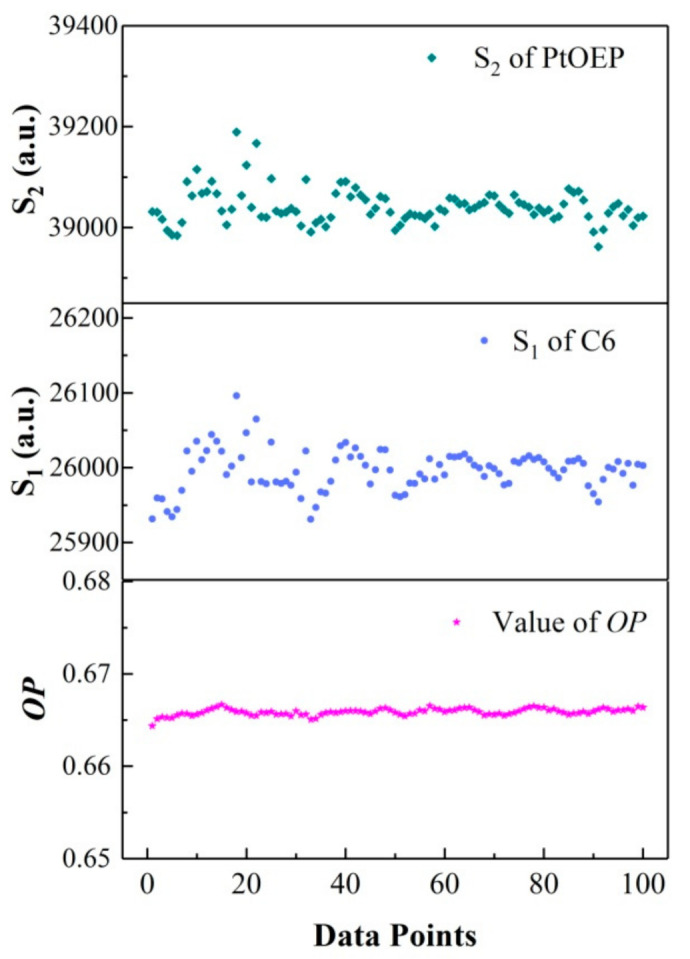
Variation in the values of *OP*, luminescence intensity of *I*_PtOEP_, and *I*_C6_.

Figure 6 shows the variation in the multiple measurement (100 times) values of *OP*, and the integration areas S_2_ of PtOEP and S_1_ of C6 under *I*_pump_ = 0.4 mW/cm^2^. As can be seen from the results, the measurement values of *OP* (~0.34%) were more stable, whereas the integration areas of PtOEP (~0.58%) and C6 (~0.64%) fluctuated with fluctuations in the excitation light significantly. This indicates that the ratiometric oxygen-sensing film can effectively eliminate the errors caused by the excitation light and fluctuation of the detector.

### 3.4. Reversibility of the Ratiometric Oxygen-Sensing System

**Figure 7:**
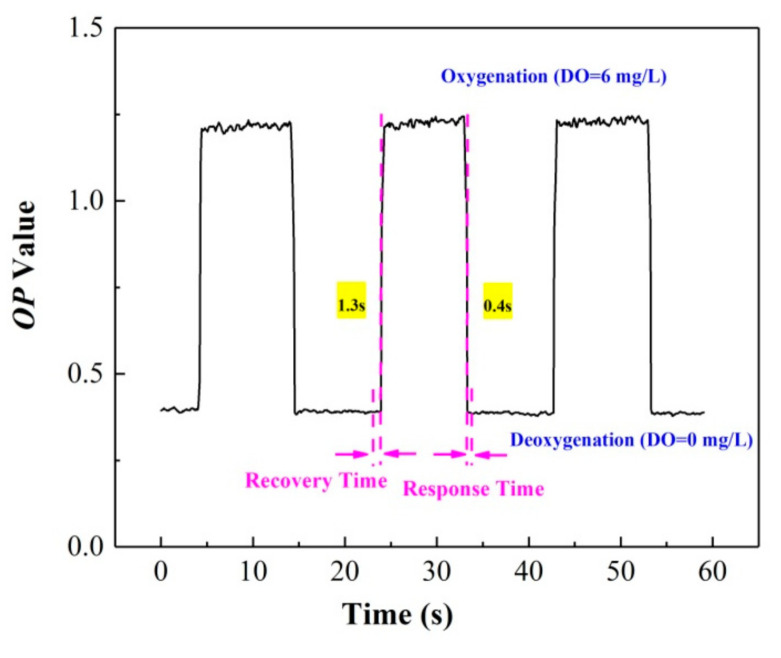
Reversibility and response time of the ratiometric oxygen-sensing system.

The reversibility is an indication of the response time and reproducibility of the ratiometric oxygen sensor. The response time includes the quenching time t_Q_ and recovery time t_R_ [29,30], which represent the time required for a 95% change in the luminescence signal; t_Q_ considers the change from an anaerobic to aerobic atmosphere, and t_R_ considers the change from an aerobic to anaerobic environment [31]. Figure 7 shows the variation of the *OP* value, and the response time of the film under the alternating conversion of the deoxygenation (DO = 0 mg/L) and oxygenation environment (DO = 6 mg/L). The results indicate that reproducible and stable luminescence signals can be obtained after testing for three cycles, and the sensor exhibits fast response times; t_Q_ is 0.4 ± 0.2 s, while t_R_ is 1.3 ± 0.2 s.

### 3.5. Detection Limit of the Ratiometric Oxygen Sensor

**Figure 8:**
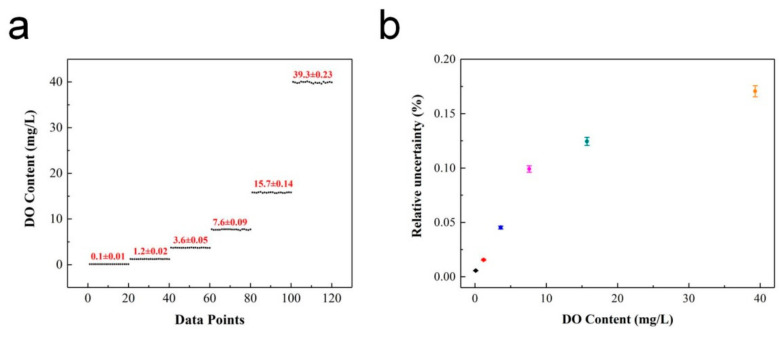
Performance of oxygen sensor: (**a**) Detection limit; (**b**) Relative uncertainty versus DO content.

The detection limit (LOD) and relative uncertainty (ΔDO/DO)% of the ratiometric oxygen sensor were obtained through further testing and analysis of the system. Samples with different DO concentrations were selected for multiple measurements. Herein, the LOD was defined as the oxygen concentration that brought the variation of the *OP* value equal to the standard deviation [23]. The relative uncertainty of the oxygen-sensing system was obtained by analyzing and comparing the difference between the measurement results and real value, which was defined as half the value of the difference between the maximum and minimum values. Figure 8a shows the multiple measurements for different DO concentration samples. From the measured *OP* values and combined with the calibration equation, the detected DO contents were 0.1, 1.2, 3.6, 7.6, 15.7 and 39.3 mg/L, respectively. The relative uncertainty during the test process was calculated as shown in Figure 8b. It can be seen that the relative uncertainty of the system increases with an increase in DO concentration, and the minimum uncertainty was 0.01 mg/L, which appeared for a DO content of 0.10 mg/L. Hence, the detection limit of the ratiometric oxygen sensor was further determined to be 0.10 (1) mg/L. For comparison, the detection limits and accuracy of different methods are listed in Table 1.

The comparison results indicate that the ratiometric oxygen sensor in this study shows a lower detection limit and better detection accuracy. The variation in relative uncertainty with the DO content was mainly attributed to the concentration ratio of the two indicators. Consequently, the performance of the ratiometric oxygen sensor can be optimized by adjusting the amounts of PtOEP and C6, which possess other minimum detection limits and uncertainties for precise detection in different water environments.

### 3.6. Investigation of DO Kinetic Process

The ratiometric oxygen sensor was applied to the investigation of the kinetic process related to the variation of the DO content in water, which was obtained based on the following calibration equation by measuring the *OP* values.

(2) $$\left[DO\right]\;=\;\left(OP-0.39\right)/0.14$$

**Figure 9:**
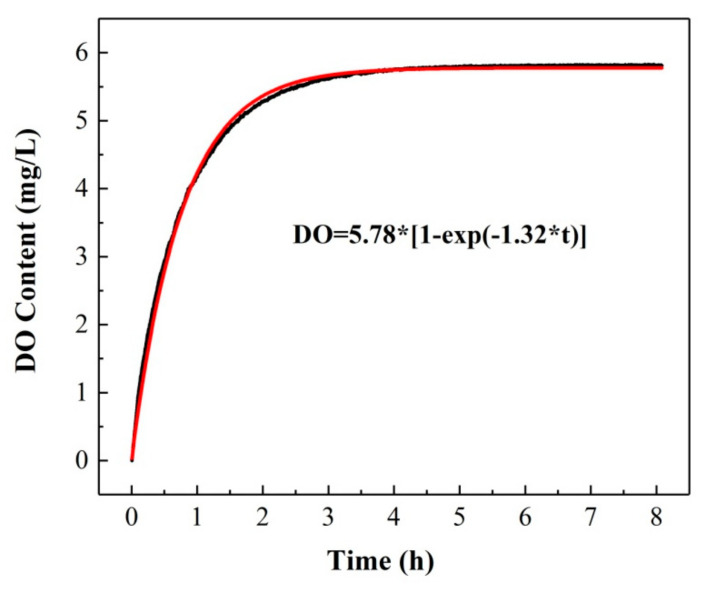
The variation of DO content; kinetic process.

Generally, the dissolution and diffusion of oxygen in water are geared to a dynamic reversible process. The saturated DO content in water under a certain condition can be obtained when the dissolution rate is equal to the diffusion rate, which is called the DO dynamic equilibrium. We selected deionized water saturated with nitrogen as the observation object to study the change in DO concentration at room temperature (T = 292.15 K). The variation of the measured DO content with time is shown in Figure 9. It can be seen that the DO content increases dramatically in the range of 1–3 h, which indicates that the gaseous oxygen molecules in the air can be dissolved in water as well. With prolonged time, the change in the DO content slackens gradually and remains stable in the time range of 4.5–7 h. Finally, the measured DO content and time satisfy an exponential function correlation relationship (RMSEC=1.08%).

(3) $$\left[DO\right]\;=5.78\;*\;\left[1-\exp\left(-1.32\;*\;t\right)\right]$$

Therefore, the saturation DO content under this condition was determined to be 5.82 ± 0.02 mg/L.

## 4. Conclusions

In summary, a ratiometric oxygen sensor based on the dynamic phosphorescence quenching mechanism with imbedded PtOEP and C6 in a poly (St-TFEMA) fluoropolymer film was developed for automatic DO content detection. Herein, PtOEP and C6 were chosen as the oxygen-sensitive and oxygen-insensitive indicators. *OP* was defined as the ratio of the luminescence integral area of C6 (S_1_) to that of PtOEP (S_2_), which was only a function of the DO concentration. The changes in the luminescence intensity of the ratiometric sensing film with DO content were investigated, demonstrating that the calibration curve between the *OP* value and DO content reveals a correlation expressed by a linear function. The automatic ratiometric oxygen detection system based on the Labview software was constructed utilizing a USB2000 spectrometer. The sensor can effectively eliminate external interference due to the variation and fluctuation of the excitation light intensity, and present good *OP* stability. The performance parameters of the ratiometric oxygen sensor were evaluated, and the detection limit was determined to be 0.10 (1) mg/L. Moreover, the oxygen sensor exhibits satisfactory reversibility, with a fast response time of 0.4 ± 0.2 s and recovery time of 1.3 ± 0.2 s. Finally, the ratiometric oxygen sensor based on the *OP*–DO calibration equation was applied to the investigation of the kinetics causing the change in the DO content in water. It was found that the DO content and time were correlated, expressed by an exponential function, and the saturation DO content at T = 292.15 K was 5.82 ± 0.02 mg/L, which appeared at *t* = 4.5 h. Furthermore, the ratiometric oxygen sensor exhibits good detection accuracy and relative uncertainty in practical application. Consequently, since the DO content detection in water samples shows good accuracy, we believe that the ratiometric oxygen sensor can be extended to other application fields.

## Figures and Tables

**Table 1:** Comparison of detection limit and accuracy of different methods.

No	Methods	Detection Limit (mg/L)	Detection Accuracy (mg/L)	Reference
1	Winkler method	0.19	0.02	[32]
2	Clark electrode method	0.1	0.1	[1]
3	Dual emission based on PtF20TPP-CdTe film	0.5	0.5	[22]
4	PtOEP-C6 ratiometric oxygen sensor	0.10	0.01	This work
